# Changes in Distress Screening and Information Reception Among Cancer Patients Following a Training for Oncology Nursing Staff: A Pilot Study

**DOI:** 10.1002/cam4.71994

**Published:** 2026-06-02

**Authors:** Lara Dreismann, Tanja Zimmermann

**Affiliations:** ^1^ Department of Psychosomatic Medicine and Psychotherapy Hanover Medical School Hanover Germany; ^2^ Department of Stem Cell Transplantation University Medical Center Hamburg‐Eppendorf Hamburg Germany

**Keywords:** cancer, cancer care, cancer nursing, communication, gastrointestinal cancer, oncology, psycho‐oncology, psychosocial, screening, visceral‐oncology

## Abstract

**Background:**

At least half of all cancer patients suffer from significant psychological distress, yet despite well‐validated screening procedures, distress often goes unrecognized or untreated. For this reason, a special training (OptiScreen‐Training) on this topic has been developed for oncology nurses and carried out on three visceral oncology wards.

**Aims:**

Investigate whether this training influences patients' psychological symptoms, experience of distress screening, informedness, receipt of recommendation, and utilization of support.

**Methods:**

To evaluate implementation, a prospective, non‐randomized pre‐post pilot study was conducted with two independent patient samples. *N* = 112 patients of the control group (CG, “care‐as‐usual”) completed questionnaires before the OptiScreen‐Training and *N* = 210 different patients of the intervention group (IG) after the training. All patients had a gastrointestinal cancer diagnosis; the majority of both samples received surgery (58.1%–79.5%) as curative treatment (61.9%–77.7%) for colon cancer (33.3%–35.7%); were male (61%–63%); and mean age was 62 years (range 21–91; SD = 11.80–13.06).

**Results:**

There was no significant difference between the CG and IG in terms of psychological distress, resources, or needs (*p* = 0.274–0.975), nor in the receipt of recommendations (*p* = 0.748) or the use of psycho‐oncological support (*p* = 0.648). In the IG, more patients indicated that they had completed a screening questionnaire (63.0% vs. 43.3%, *p* = 0.001) and received psycho‐oncology information material (68.3% vs. 46.2%; *p* < 0.001).

**Conclusion:**

Following the OptiScreen‐Training more patients reported they had been screened and informed, suggesting nursing staff are applying it in practice. However, it did not affect support use or distress levels, highlighting that screening alone isn't enough to improve psycho‐oncological care.

## 1 Background

About 50%–65% of patients with cancer report significant psychological distress and, on average, eight different burdensome problems such as fatigue, sleeping problems, and anxiety [1, 2]. At the same time, a significant proportion of patients do not feel well informed about psycho‐oncological support services [3], despite the expansion of support services and availability of information material in recent decades. A gap persists between patients requiring psycho‐oncological support and those using services. This discrepancy is partly due to challenges in the identification of distress and provision of personalized information and referrals to psycho‐oncology. The lack of psycho‐oncological support in crisis situations can have long‐term consequences. It is well known that one‐third of all cancer patients develop a mental disorder, with adjustment disorders, depression, or anxiety disorders being the most prevalent [4]. Although all patients can experience distress, specific cancers—like gastrointestinal cancers—pose distinct challenges. Some evidence suggests higher distress levels in these cases despite limited research [1]. For instance, patients diagnosed with colorectal cancer who receive a stoma report a decline in body image satisfaction, which increases over time [5]. A recent study of gastrointestinal cancer patients revealed that about half of all patients suffered from anxiety, which decreased after discharge [6]. Another study demonstrated that about 20% of patients with these types of cancer fulfill the criteria for a mental disorder and continue to exhibit symptoms of depression and anxiety even 6 months following surgery [7]. Apart from these results, there is a lack of knowledge regarding the particular psychological distress in patients treated in visceral oncology cancer centers, their experience with screening, and their need for information and support.

Psycho‐oncology interventions can effectively reduce distress and improve quality of life [8]. Therefore, patients who require psycho‐oncological treatment need to be identified, informed and referred to individualized support. According to guidelines and recommendations such as those of the National Comprehensive Cancer Network, screening questionnaires are the right tool to identify significant distress and to ask patients about their need for psycho‐oncological support [9]. However, the implementation of screening procedures is not carried out properly in practice, for example, the absence of explanation during provision of screening questionnaires, inadequate collection, lack of evaluation, failure to communicate the result or insufficient patient information regarding available support options [10, 11, 12]. The challenges inherent in the implementation of screening procedures result in distressed patients not receiving adequate psycho‐oncological care. The causes for this issue are multifaceted and vary according to the circumstances but factors such as lack of time, missing resources, protocols and responsible persons or a low priority of psycho‐oncology can contribute to the failure in distress screening implementation [13, 14]. Another potential explanation is the uncertainty and lack of information experienced by medical staff themselves [14, 15]. A previous study conducted with nursing experts identified the need for training in psycho‐oncological screening and showed that medical teams often felt unprepared to address related issues [14, 16]. At the same time, the referral by oncology staff is a good predictor of the actual utilization of psycho‐oncological support [17, 18]. Nevertheless, if patients do not feel well informed about the contents of psycho‐oncology, this may be a reason to decline support [19]. One approach to overcoming these barriers and insecurities associated with the screening process is to educate the staff who work most closely with patients: oncology nurses. The National Institute for Health and Care Excellence also recommends that nurses be responsible for conducting screening and measuring the psychological distress of their patients [20]. In particular, communication training improves awareness and identification of distress in patients [21], reduces feelings of overwhelmed and insecure [22], increases self‐confidence, self‐efficacy and communication skills [23, 24, 25]. As indicated in the literature, benefits for patients entail: more satisfaction, reduced distress and improved coping strategies [26, 27]. The curricula for nursing education and specialized training in Germany vary from region to region and do not include psycho‐oncology or screening, which makes additional training essential. Therefore the “Optimized psycho‐oncological care through an interdisciplinary care algorithm‐from screening to intervention” (OptiScreen)‐Study developed a training intervention for nurses (OptiScreen‐Training) with a focus on the communication process and screening procedure [28]. The training was already evaluated as successful from the nursing perspective in terms of satisfaction, feasibility and acceptance as well as an reducing insecurities regarding the screening process [25]. The aim of this current paper is to evaluate implementation of the OptiScreen‐Training in regard to patient outcomes (a) primary outcomes: psychological symptoms (b) secondary outcomes: distress screening experience, informedness, receipt of recommendation and (c) additional outcome: utilization of psycho‐oncological support [28].

## 2 Methods

### 2.1 Study Design and Procedure

**FIGURE 1:**
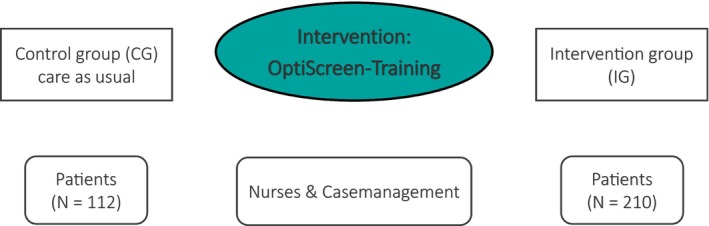
Study design OptiScreen.

The OptiScreen‐Study was designed to evaluate implementation as a prospective, non‐randomized pre‐post pilot study. The intervention comprises a training course for nursing staff on screening and psycho‐oncology (“OptiScreen‐Training”) on three pilot wards of the visceral oncology center of the Hannover Medical School. According to the internal hospital guidelines concerning distress screening, this procedure is to be conducted at the time of admission to the hospital by nurses or case management. In case of significant distress and/or wish for psycho‐oncological support, a referral to psycho‐oncology is initiated. The study population included two independent cancer patient samples from the three participating wards, who underwent treatment prior to and following the training. As a care as usual condition, patients (control group; CG) completed a questionnaire package during their inpatient stay before the OptiScreen‐Training for nurses was carried out on the wards. After the OptiScreen‐Training had been carried out, different patients (intervention group; IG) were again examined during their inpatient stay using the questionnaire package (see Figure 1).

**FIGURE A1:**
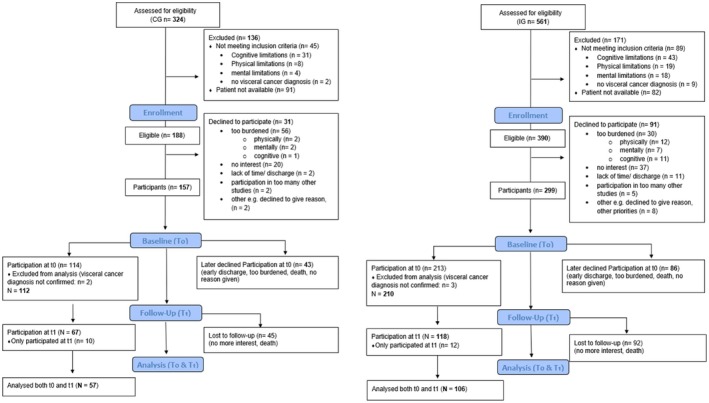
Flowchart of the control and intervention group. CG, Control Group; IG, Intervention Group.

The study was conducted during the Covid‐19 pandemic, which required special caution and adequate recruitment routines. The participants of the CG were recruited between May 2020 and January 2021 (9 months) before the intervention (OptiScreen‐Training), the recruitment of the IG started 3 months after the intervention (OptiScreen‐Training) to limit direct training bias and was conducted between June 2021 and October 2022 (17 months). The recruitment phases had to be adapted to local restrictions on study staff and patient contact during the Covid‐19 pandemic, resulting in varying lengths for the corresponding targeted sample sizes. Patients who met the inclusion criteria (a) diagnosis of a malignant solid tumor associated with visceral oncological disease: intestine, pancreas, stomach, liver, esophagus; (b) a minimum age of 18 years; (c) cognitive ability to consent to study participation were approached personally by the study staff during their first 2 weeks after admission. Exclusion criteria were accessed (severe physical, cognitive, and/or language limitations for example, unable to complete questionnaires in German) and patients informed about the study prospects (see Figure A1). After filling out a consent form (in accordance with the Declaration of Helsinki), each participant filled out the questionnaire, which was collected during their stay. Patients who declined the participation (non‐participants) were asked to answer a short oral interview on why they declined and their current experience with psycho‐oncology and socio‐demographic as well as medical information. Positive ethic vote has been received from Hannover Medical School (8478_BO_K_2019).

### 2.2 Intervention

The OptiScreen‐Training [29] is a comprehensive training concept designed to equip nurses with the necessary knowledge regarding distress screening and psycho‐oncological services. The training focuses on the implementation of screening procedures in combination with personalized information for patients on available support options and recommendations tailored to their individual needs. The training reduces personal barriers and uncertainties among nurses with regard to screening, thereby supporting communication between patient and nurse. The training consists of three modules, with a duration of 1.5–2 h each, delivered on three separate dates. The modules include “Psycho‐oncology and Mental Disorders in Cancer Patients”, “Psychological Distress and Screening Methods” and “Communication in the Screening Process and Self‐care”. The training entails a variety of educational methodologies including presentations, case studies, videos, exercises, and role‐playing. The training was conducted by psycho‐oncologists and is based on practical observations as well as theory‐ and literature‐driven research and nursing experts' opinions in the context of a qualitative needs and hurdle analysis through interviews [16, 25, 30]. An additional 1‐h refresher training session for two of the three participating wards was conducted 1 year after initial training (March 2022).

### 2.3 Participants

The CG consists of *N* = 112 patients (37.5% women), the IG of *N* = 210 participants (36.7% women; see Table 1). The average length of stay was 14.27 days (SD = 16.38) for the CG and 11.77 (SD = 15.92) for the IG. The mean age was 61 years in both groups and the most frequent tumor entity was colon cancer with the majority receiving surgery with a curative treatment goal. Groups differed regarding the prevalence of metastases, current medical treatment, treatment goal, and tumor location.

**TABLE 1:** Demographic and cancer‐related characteristics of the sample.

	CG (*N* = 112)	IG (*N* = 210)	Difference test
	*M* (SD)	*M* (SD)	
Age in years	61.87 (11.80)	61.67 (13.06)	*t*(320) = 0.141, *p* = 0.888
Range	25–88	21–91	
Months since initial diagnosis	16.92 (39.42)	16.22 (29.61)	*t*(314) = 0.179, *p* = 0.858
Range	0–243	0–170	

	*n* (*%*)^a^	*n* (*%*)	
*Tumor location*			
Anal	0 (0)	0 (0)	*χ* ^2^(6) = 14.35, *p* < 0.05
Pancreas	10 (8.9)	15 (7.1)	
Colon	40 (35.7)	70 (33.3)	
Bilde duct	19 (17.0)	27 (12.9)	
Liver	25 (22.3)	60 (28.6)	
Stomach	2 (1.8)	10 (4.8)	
Esophagus	16 (14.3)	15 (7.1)	
Other	0 (0)	12 (5.7)	
*Metastases*			
Yes	38 (33.9)	81 (38.6)	*χ* ^2^(1) = 7.47, *p* < 0.01
No	56 (50.0)	57 (27.1)	
*Treatment goal*			
Curative	87 (77.7)	130 (61.9)	*χ* ^2^(4) = 10.56, *p* < 0.05
Palliative	7 (6.3)	32 (15.2)	
Supportive	1 (0.9)	8 (3.8)	
Unaware	10 (8.9)	26 (12.4)	
*Current medical treatment* ^a^			
Surgery	89 (79.5)	122 (58.1)	χ^2^(1) = 14.77, *p* < 0.001
Chemotherapy	23 (20.5)	40 (19)	*χ* ^2^(1) = 0.10, *p* = 0.749
Radiotherapy	6 (5.4)	6 (2.9)	*χ* ^2^(1) = 1.27, *p* = 0.259
Radiochemotherapy	2 (1.8)	6 (2.9)	*χ* ^2^(1) = 3.46, *p* = 0.556
Immunotherapy	4 (3.6)	20 (9.5)	*χ* ^2^(1) = 3.75, *p* = 0.053
Other	20 (17.9)	49 (23.3)	*χ* ^2^(1) = 1.30, *p* = 0.254
*Sex*			
Female	42 (37.5)	77 (36.7)	*χ* ^2^(1) = 0.06, *p* = 0.814
Male	68 (60.7)	132 (62.9)	
Other	0	0	
*Relationship status* ^a^			
Single	8 (7.1)	25 (11.9)	*χ* ^2^(1) = 1.80, *p* = 0.180
In a relationship/married	81 (72.3)	153 (72.9)	*χ* ^2^(1) = 0.01, *p* = 0.918
Divorced	11 (9.8)	20 (9.5)	*χ* ^2^(1) = 0.01, *p* = 0.931
Separated	3 (2.7)	4 (1.9)	*χ* ^2^(1) = 0.21, *p* = 0.650
Widowed	11 (9.8)	13 (6.2)	*χ* ^2^(1) = 1.40, *p* = 0.237
*Children*			
Yes	88 (78.6)	152 (72.4)	*χ* ^2^(1) = 1.48, *p* = 0.225
No	24 (21.4)	58 (27.6)	
*Nationality*			
German	102 (91.1)	194 (92.4)	*χ* ^2^(1) = 0.42, *p* = 0.519
Other	6 (5.4)	8 (3.8)	
*Highest educational degree*			
≤ 9 years of school	30 (26.8)	47 (22.4)	*χ* ^2^(5) = 3.66, *p* = 0.599
10 years of school	33 (29.5)	65 (31)	
Advanced technical college certificate	17 (15.2)	31 (14.8)	
Baccalaureate	29 (25.9)	59 (28.1)	
Without degree	1 (0.9)	1 (0.5)	
Other	0 (0)	5 (2.4)	
*Vocational training*			
Apprenticeship	56 (50)	94 (44.8)	*χ* ^2^(5) = 6.05, *p* = 0.302
Technical College	14 (12.5)	29 (13.8)	
University	20 (17.9)	41 (19.5)	
Without vocational training	4 (3.6)	7 (3.3)	
Other	12 (10.7)	30 (14.3)	
*Employment status*			
Full time	33 (29.5)	60 (28.6)	*χ* ^2^(7) = 7.02, *p* = 0.427
Part‐time	9 (8)	13 (6.2)	
Unemployed	7 (6.3)	7 (3.3)	
Retired/superannuated	44 (39.3)	78 (37.1)	
Reduction in earning capacity	6 (5.4)	28 (13.3)	
Housewife/‐husband	3 (2.7)	5 (2.4)	
In training/retraining	0 (0)	1 (0.5)	
Other	6 (5.4)	9 (4.3)	
*Household income in €/month*			
< 500€	1 (0.9)	2 (1)	*χ* ^2^(7) = 8.38, *p* = 0.300
< 1500€	17 (15.2)	38 (18.1)	
< 2500€	40 (35.7)	48 (22.8)	
< 3500€	16 (14.3)	48 (22.9)	
> 3500€	26 (23.2)	50 (23.8)	

*Note:* *N* because of missings and dual display partly divergent.

Abbreviations: CG, control group; IG, intervention group.

^a^ Multiple answers possible.

### 2.4 Measures

Sociodemographic data, medical information, and all items were self‐reported by the participants as part of the questionnaire package. The questionnaire consisted of different validated instruments and additional scales on distress screening experience (see Table 2).

**TABLE 2:** Frequencies of cut‐offs within CG and IG.

Variable/scale	Categories/cut‐offs	CG % (*n*)	IG % (*n*)
DT	≥ 5 (significant)	59.5 (66)	51.5 (106)
CDT	≥ 5 (significant)^a^	46.8 (51)	30.4 (62)
PHQ‐9	≥ 5 (mild)	30.6 (30)	33.3 (66)
	≥ 10 (moderate)	17.3 (17)	20.2 (40)
	≥ 15 (moderately severe)	9.2 (9)	9.1 (18)
	≥ 20 (severe)	3.1 (3)	2.0 (4)
GAD‐7	≥ 5 (mild)	22.8 (23)	24.2 (47)
	≥ 10 (moderate)	5.0 (5)	8.2 (16)
	≥ 15 (severe)	4.0 (4)	4.1 (8)
FoP‐Q‐SF	≤ 23 no/low	30.0 (27)	31.5 (53)
	≥ 24 moderate	42.2 (38)	37.5 (63)
	≥ 34 high	27.8 (25)	31.0 (52)
BIS	≥ 10	23.5 (23)	24.6 (48)

*Note:* *N* partly reduced due to missing values for cut‐off calculation, only valid cases reported CG *N* = 90–112; IG *N* = 168–210.

Abbreviations: BIS, Body Image Scale; CG, Control Group; Covid‐Distress‐Thermometer DT; DT, Distress Thermometer; FoP‐Q‐SF, Fear of Progression Questionnaire; GAD‐7, Generalized Anxiety Disorder Scale; IG, Intervention Group; PHQ‐9, Patient Health Questionnaire.

^a^ Cut‐Off not validated.

#### 2.4.1 Primary Outcomes

*Psychosocial distress* was measured with the German version of the NCCN Distress Thermometer (DT, [31]), which consists of a single item asking the respondent to estimate their global level of cancer‐related distress in the past week on a Likert scale of 0 (no distress) to 10 (extreme distress). A cut‐off value of 5 or higher indicates significant psychosocial distress.

In addition to the original DT, we added another complementary adaptation of the DT to measure the psychosocial burden in face of the Covid‐19 pandemic (Corona Distress Thermometer, CDT) during the study phase. The item was adapted to “Please circle the number that best describes how much distress you have been experiencing in the past week, including today *in regard to the Covid‐19 pandemic*”. Answering options and cut‐off value were used based on the DT but not yet validated.

*Depressiveness* was measured using the German version of the Patient Health Questionnaire (PHQ‐9) [32] which uses nine items to assess the symptoms of depression according to DSM‐IV. Patients rate on a four‐point Likert scale from 0 (not at all) to 3 (almost every day) how often they have felt affected by the respective symptoms in the last 2 weeks. There are cut‐off values for mild, moderate, moderate–severe, and severe depression at 5, 10, 15, and 20. In the present samples, the internal consistency was *α* = 0.85–0.86.

*Anxiety* was measured with the German version of the Generalized Anxiety Disorder Scale (GAD‐7, [33]) with seven items. Patients indicate how often they have felt affected by each symptom in the last 2 weeks. The items are answered on a four‐point Likert scale ranging from 0 (not at all) to 3 (almost every day). 5, 10, and 15 represent cut‐off values for mild, moderate, and severe anxiety symptoms respectively. In both groups, the internal consistency was *α* = 0.90.

*Fear of Cancer Reoccurrence* was measured with the German version of the short form of the Fear of Progression Questionnaire (FoP‐Q‐SF, [34]) which consists of 12 items. The items are answered on a five‐point Likert scale ranging from 1 (never) to 5 (very often). A cut‐off of 34 indicates dysfunctional fear of cancer reoccurrence, while values between 0–23 indicate low and 24–33 moderate fear [35]. The present samples also showed a high internal consistency (Cronbach's *α* = 0.90).

*Health literacy* was assessed with the German version of the European Health Literacy Survey Questionnaire (HLS‐EU‐Q16, [36]) with 16 items, rated on a four‐point Likert scale from 1 (very difficult) to 4 (very easy), with higher scores indicating better health literacy. Dichotomized scores can be categorized into 1–8 (problematic); 9–12 (problematic); and 13–16 (adequate) health literacy scores. Internal consistency ranged between *α* = 0.88–0.90.

*Social support* was measured with the German version of the “Scales for Social Support in Illness” (SSUK‐8) with 8 items [37]. The SSUK‐8 comprises two subscales (stressful and positive aspects of interpersonal interactions), each with four questions that can be answered with 0 (never) and 4 (always). Internal consistency was high both for the positive support (Cronbach's *α* = 0.84) and for the negative interaction subscale (Cronbach's α = 0.75–0.80).

*Quality of life* was assessed with the German version of the Short‐Form Health Survey (SF‐8) [38]. The 8 items are rated from 1 to 5 or 1 to 6 with different answering categories, while higher scores indicate better quality of life. As two subscales, physical (PCS) and mental component summary scores (PCS), are calculated by weighting each item with a norm‐based scoring method. Internal consistency ranged in the participant groups between *α* = 0.89–0.90.

*Body image* was measured with the German Body Image Scale [39]. The 10 items are used to measure affective, behavioral and cognitive body image symptoms in the last week, which can be rated on a four‐point Likert scale from 0 (not at all) to 3 (very much). A cut‐off value of ≥ 10 can be used to describe a low body image satisfaction [40]. The BIS has an internal consistency of *α* = 0.88–0.86 in the present samples.

#### 2.4.2 Secondary Outcomes

*The OptiScreen‐Questions* were developed as exploratory process indicators specifically for the OptiScreen‐Study [28] in order to investigate patients' experience with distress screening and psycho‐oncological support (see Table A1). The questions of whether information material (e.g., flyers), the distress screening (questionnaire), a recommendation for support, or psycho‐oncological support was received can be answered with “yes” vs. “I don't know”, “no”, while the last categories were combined for analysis. Questions regarding the quality of support, the respondent's own level of information, or satisfaction with the comprehensiveness of the information (additional outcomes) can be rated on a five‐point Likert scale from 1 (not at all) to 5 (very).

### 2.5 Statistical Analysis

Statistical analyses were performed using SPSS for Windows [41]. Means, standard deviations, and frequencies were calculated for descriptive evaluation and sample characteristics as well as internal consistency for both the CG and IG separated. Prior to analysis, all data were screened for implausible or out‐of‐range values. Cases with missing items exceeding the thresholds specified in the respective scoring manuals were excluded from the corresponding descriptive scale analyses. Statistical assumptions for all analyses were verified prior to testing. If validated cut‐off values for the primary outcomes were available, frequencies for each category and group were calculated. Differences before and after the training between the two different groups were calculated using unpaired *t*‐tests or chi‐squared tests. No imputation of missing values was performed. All tests were two‐sided, and the significance level was set at 0.05. We used the outcome of actual uptake of psycho‐oncological support as an outcome variable to calculate predictors (both based on literature and theoretical considerations on clinical relevance of the OptiScreen‐Questions) with a binomial logistic regression. Nagelkerke *R* ^2^ was calculated to explain the variance. To address multicollinearity, variance inflation factors (VIF) were examined prior to model fitting and all VIF values were < 5.

## 3 Results

### 3.1 Sample Characteristics

#### 3.1.1 Participants vs. Non‐Participants

Compared to non‐participants (NP), the study participants in the CG were younger (CG: *M* = 62 years vs. NP *M* = 67 years; *t*(141) = 2.181, *p* < 0.05, *d* = 0.43), and in the IG older (IG: *M* = 62 years vs. NP *M* = 56 years; *t*(299) = −0.830, *p* = 0.407). There was no difference in gender distribution in both groups (CG: *χ* ^2^(1) = 1.618, *p* = 0.203; IG: *χ* ^2^(1) = 0.392, *p* = 0.531). NP had a higher frequency of receiving curative cancer treatment compared to participants (NP 23.8% vs. CG 9.6%, *χ* ^2^(1) = 30.495, *p* < 0.001, *φ* = 0.547; NP 33.0% vs. IG 13.3%; *χ* ^2^(1) = 43.453, *p* < 0.001, *φ* = 0.467). There was no significant difference in distress (DT) between NP and study participants (CG: *t*(137) = 0.558, *p* = 0.578; IG: *t*(294) = 1.096, *p* = 0.274).

### 3.2 Primary Outcomes

#### 3.2.1 Psychological Symptoms

The majority of participants (except for DT) in both groups did not show any symptoms that were categorized as severe or high above the official cut‐off values (See Table 2).

There are no significant differences between CG and IG regarding psychological distress, symptoms, resources or needs, except for distress due to Covid (see Table 3).

**TABLE 3:** Means and differences of psychological symptoms between CG and IG.

	CG, *M* (SD)	IG, *M* (SD)	df	*t* test	*p*
*Scale/Symptom*					
Psychosocial Distress (DT)^a^	5.06 (2.63)	4.71 (2.81)	315	1.094	0.275
Distress due to Covid (Covid‐DT)^a^	4.38 (2.60)	3.23 (2.51)	311	3.818	< 0.001
Depression (PHQ‐9)^b^	7.17 (5.30)	7.40 (5.27)	294	−0.354	0.724
Anxiety (GAD‐7)^b^	3.70 (3.96)	4.36 (4.45)	293	−1.249	0.213
Physical QOL (SF‐8)^c^	39.65 (11.60)	38.77 (12.53)	302	0.592	0.554
Mental QOL (SF‐8)^c^	43.41 (11.67)	44.09 (11.51)	302	−0.484	0.629
Fear of cancer recurrence (FoP‐Q‐SF)^d^	29.27 (10.00)	28.74 (9.86)	256	0.756	0.451
Health Literacy (HLS‐EUQ16)^e^	12.14 (3.30)	12.60 (3.26)	280	−1.102	0.271
Positive Social Support (SSUK)^f^	13.98 (3.05)	13.73 (3.15)	292	0.660	0.510
Negative Social Support (SSUK)^f^	5.28 (3.87)	4.58 (3.60)	298	1.544	0.124
Body Image (BIS)^b^	5.89 (6.25)	6.03 (6.34)	291	−0.176	0.860

*Note:* *N* partly reduced due to missing values.

Abbreviations: CG, Control Group; df, degrees of freedom; IG, Intervention Group; M, Mean; *N* CG, 90–103; *N* IG, 168–201; SD, standard deviation.

^a^ *Min* = 0; *Max* = 10.

^b^ *Min* = 0; *Max* = 3.

^c^ *Min* = 1; *Max* = 6.

^d^ *Min* = 1; *Max* = 5.

^e^ *Min* = 1; *Max* = 4.

^f^ *Min* = 0; *Max* = 4.

### 3.3 Secondary Outcomes

#### 3.3.1 Experience With Distress Screening

**FIGURE 2:**
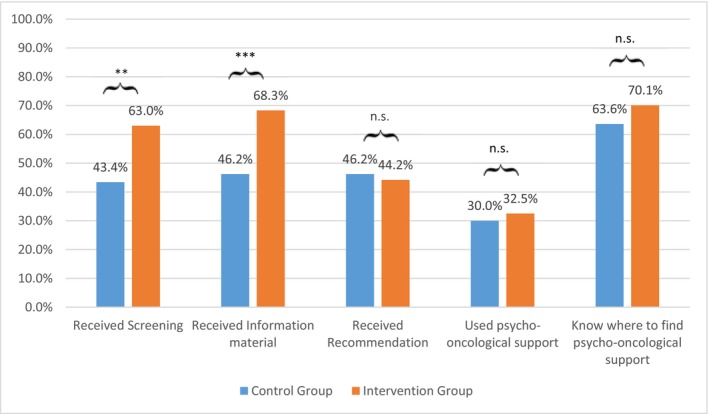
Frequencies of screening experience in control and intervention group. **p = 0.001; ***p < 0.001; n.s., not significant, *N* CG = 104–110; *N* IG = 199–203, *N* partly reduced due to missing values.

Results show a significant effect between CG and IG for receiving the distress screening (*χ* ^2^(1) = 10.817, *p* = 0.001, *φ* = 0.18) and receiving information material (*χ* ^2^(1) = 14.147, *p* < 0.001, *φ* = 0.22), in relation to the fact that more participants of the IG reported being screened and received information material more often. The rating whether the information material was comprehensive (CG *M* = 4.00; SD = 0.68; IG *M* = 4.02; SD = 0.74) was not significantly different between groups (*t*(169) = −0.186, *p* = 0.600). There was no significant difference in feeling well informed about support services (CG *M* = 2.90; SD = 1.09; IG *M* = 3.23; SD = 1.14) between both groups (*t*(300) = −2.497, *p* = 0.433). No significant differences were reported for receiving a recommendation on support services (*χ* ^2^(1) = 0.103, *p* = 0.748), knowing where to find support (*χ* ^2^(1) = 1.316, *p* = 0.251), and actual uptake of psycho‐oncological support (*χ* ^2^(1) = 0.208, *p* = 0.648) or social‐legal advice (*χ* ^2^(1) = 0.035, *p* = 0.851) or pastoral care (*χ* ^2^(1) = 0.392, *p* = 0.531) or self‐help groups (*χ* ^2^(1) = 0.361, *p* = 0.548). The frequencies of participants who reported “yes” are shown in Figure 2.

### 3.4 Further Outcomes

#### 3.4.1 Uptake of Psycho‐Oncological Support

Since there was no difference between groups in the utilization of psycho‐oncological support, the following model was calculated using gender, receiving recommendation of support, receiving distress screening and receiving information material as predictors for the uptake (yes/no) for both groups together. The model accounted for 22.3% of the variance. Of the four variables included in the model, three were significant: gender (*p* < 0.05), receiving recommendation of support (*p* < 0.001), and receiving information material (*p* < 0.05). Receival of the distress screening had no significant effect (*p* = 0.677). Referral had a positive effect on uptake, with odds ratio (OR) of 3.855 (95% CI [2.176, 6.829]), as well as receival of informational materials, with OR of 2.075 (95% CI [1.053, 4.090]). Women were more likely than men to use psycho‐oncological support with OR of 0.555 (95% CI [0.320, 0.961]).

There was no significant difference between the two groups in terms of current desire for psycho‐oncological support (*χ* ^2^(1) = 0.642, *p* = 0.423) or previous experience with psychological support/psychotherapy prior to cancer diagnosis (*χ* ^2^(1) = 1.320, *p* = 0.251).

## 4 Discussion

The aim of the present study was to evaluate whether training nursing staff in psycho‐oncological topics and screening for psychosocial distress was implemented successfully by taking patient reported outcomes regarding psychological symptoms, distress screening experience, informedness, receipt of recommendation, uptake of support into account. Patients in the intervention group were significantly more likely to report receipt of distress screening (secondary outcomes) after nursing training than patients in the control group (before training). Obviously, the content of the OptiScreen‐Training on the screening procedure, such as which screening instrument is given to patients when and how, or how nurses can respond to patients' questions, contributed to the fact that screening was reported by patients to be carried out more frequently after the training than before. Taking the small to medium effect sizes into account, training on screening procedures appears to be a useful tool for improving the screening routine.

The report of psychological symptoms (primary outcomes) did not differ in the CG before the training compared to the IG after the training. The exception is the distress due to Covid which decreased in the intervention group. The lifting of local hygiene restrictions and risk mitigation measures during the recruitment period should be considered as contributing factors in this regard. Overall, the distress levels of the present sample of patients with visceral cancers appear to be comparable with other cancer entities and studies with German cancer patients [2]. The lack of impact of the nursing training on the psychological symptoms could be because the OptiScreen‐Training primarily focuses on the screening process rather than on communication training that directly aims to reduce distress or improve coping skills [27]. A study comparing trials and direct effects of screening on psychological distress found divergent results [42]. Asking about distress and taking an interest in it (also through a questionnaire) can have a positive effect on distress [43]. At the same time, other studies show that this alone is not sufficient and that appropriate psycho‐oncological care must be initiated [12]. Our findings support this argument that reliable care pathways and algorithms are needed, in which screening and appropriate needs‐based psycho‐oncological interventions should be embedded.

As a result of the training, nursing staff became better informed about psycho‐oncology [25], which might correlate with the increase in the distribution of information material to patients after the training (part of secondary outcomes). The training also focuses on imparting specific practical knowledge, for example, how flyers can be ordered. The results indicate that this could be well implemented by the nursing staff in their daily clinical routine. Since earlier studies show that 60% of patients do not feel well informed about psycho‐oncological services, the result of this study, that 68% of patients now receive information material on this topic after training, is an important contribution to better care [3]. However, around 70% of patients already knew where to find psycho‐oncological support both before and after the training. This could be due to the fact that low‐threshold information (posters, website, fellow patients, etc.) was already available on the wards.

However, even well‐informed patients do not necessarily make use of support [44]. This is also evident in the present study, in which the training of nursing staff did not impact the utilization of psycho‐oncological support services by patients (additional outcome). One reason could be that this is not yet a priority during the stay in the acute hospital, especially as surgical treatment is the main focus for visceral oncology patients [45]. Findings also show that well‐informed patients are already relieved to know where they can get outpatient help after their hospital stay [44]. Conversely, patients who feel less well‐informed show a higher level of distress [46, 47]. Patients did not report an increase of recommendations on support services. This may be explained by the nurses' own definition of their role in cancer care and whether or not this includes giving recommendations on outpatient care. Nevertheless, the present study was able to demonstrate that the receipt of information and recommendation of psycho‐oncology support and being female have a positive predictive effect on utilization, a finding that aligns with previous studies [48, 49]. The Screening itself did not have a significant impact on the uptake of support and therefore should be combined with a personal recommendation. Despite the training having been found to reduce personal barriers such as insecurities, the structural issues for example, time constraints, lack of compensation for screening and short inpatient stays persisted. These structural barriers may have prevented referrals and thus limited patient access to psycho‐oncological support [14].

### 4.1 Clinical Implications

Even if training can improve the recognition of psychological distress, valid screening is important, as otherwise the medical team often misjudges distress [21, 50]. As recommended in the guideline by the National Comprehensive Cancer Network [9], all cancer patients should be screened for distress, which means regardless of the clinical impression. Nevertheless, the process and implementation in clinical care are not always optimal. In particular, the responsibilities associated with screening in addition to its implementation are often challenging in routine care settings. The present study results indicate that training nurses represents one possible way to optimize screening routines, which may in turn contribute to higher screening rates and increased provision of information material to patients. The current findings may therefore serve to emphasize the potential effects of being informed and receiving screening regardless of psychological distress, given that the two study groups did not appear to differ significantly in terms of levels of distress.

### 4.2 Limitations

The results need to be considered in view of various limitations. As this study was designed as a prospective, non‐randomized pre‐post pilot study, the analyses were primarily descriptive rather than confirmatory. Accordingly, no adjustment for baseline differences (e.g., treatment goal or metastatic status) was performed in the key outcome analyses. This potential limitation may affect the comparability of the two groups and could introduce potential confounding factors, which should be considered when interpreting the findings. No formal correction for multiple comparisons was applied, accordingly, the risk of Type I error cannot be excluded. All results should be interpreted with caution and await replication in randomized controlled trials. Few patients may have reported not being screened or using psychosocial support, but may have received both just after participating in the study. Therefore, an additional follow up was conducted 3 months later, which has yet to be analyzed. Moreover, the levels of distress during the 18‐month recruitment period may have been influenced and varied by different stressors to the Covid‐19 pandemic (restrictions, fear of infection, different visitor regulations at the hospital etc.). Bias cannot be ruled out, as the Covid related burden was only assessed using the newly adapted CDT as one screening item. Also, the fluctuation of nursing staff on the three relevant wards was not systematically observed but due to the staff shortage it can be expected that the number of trained nurses on each ward has decreased over the time of the study [51]. The incomplete documentation of medical data in the hospital system was also a limiting factor in the analysis. Furthermore, the self‐developed OptiScreen questions on experience with screening and psycho‐oncology need to be validated and are therefore to be interpreted as process indicators.

### 4.3 Perspectives for Future Research

The results of the study show that it seems useful to continue training for nurses in relation to psychological symptoms, the experience and seeking of support. Future studies should also examine other types of cancer and treatment settings. In addition, a longer period of observation to assess long‐term effects for both nurses and patients would be useful in future studies. Moreover, a clustered, randomized controlled trial would be the appropriate design to generate robust evidence following this first pilot study. Investigating the impact of booster training and methods of reinforcement might also be useful to see how training effects can be sustained.

## 5 Conclusions

More patients reported the receipt of screening and psycho‐oncological information material from trained nurses after OptiScreen‐Training. This can be seen as an important step towards optimizing screening in clinical routine. Since screening alone is not enough to reduce psychological distress, it is unsurprising that no differences in psychological symptoms were reported by patients before vs. after training and that any changes may also be subject to time effects. Nevertheless, better informed patients can lead to a higher utilization of psycho‐oncological care, which can then contribute to a reduction in psychological distress. This study shows that training for nurses can be an important aspect of improving psycho‐oncological care.

## Author Contributions

**Tanja Zimmermann:** conceptualization, funding acquisition, writing – review and editing, project administration, supervision, resources, validation. **Lara Dreismann:** methodology, formal analysis, investigation, writing – original draft, visualization, data curation, conceptualization.

## Funding

This work was supported by German Cancer Aid (Deutsche Krebshilfe, 70113550, 2020).

## Ethics Statement

Positive ethic vote has been received from Hannover Medical School (8478_BO_K_2019).

## Conflicts of Interest

The authors declare no conflicts of interest.

## Data Availability

The data that support the findings of this study are available on request from the corresponding author. The data are not publicly available due to privacy or ethical restrictions.

## Appendix

**TABLE A1:** OptiScreen questions and item examples.

Scale/answering format	Item
*Screening and information (6 items)*	
Yes vs. No/I don't know	“Have you received a questionnaire regarding your mental distress?”
	“Have you received information material (e.g., flyer or brochure) on psycho‐oncological support services”
	“Have you received a recommendation on support services from your doctor or nurses?”
	“Do you know where you can find psycho‐oncological support?”
1 (not at all) to 5 (very)	“If you received information material, how comprehensive was it?
	“How well informed do you currently feel about psycho‐oncological support services?”
*Support (5 items)*	
Yes vs. No/I don't know	“Have you previously used psycho‐oncological or psychotherapeutic support?”
	“Do you currently wish to talk to a psycho‐oncologist?”
	“Have you used social‐legal advice during your current inpatient stay?”
	“Have you used self‐help‐group support during your current inpatient stay?”
	“Have you used pastoral care during your current inpatient stay?”
